# Nitrogen Deficiency-Induced Decrease in Cytokinins Content Promotes Rice Seminal Root Growth by Promoting Root Meristem Cell Proliferation and Cell Elongation

**DOI:** 10.3390/cells9040916

**Published:** 2020-04-09

**Authors:** Qi Wang, Yanchun Zhu, Xiao Zou, Fengfeng Li, Jialiang Zhang, Ziyi Kang, Xuefei Li, Changxi Yin, Yongjun Lin

**Affiliations:** 1 College of Plant Science and Technology, Huazhong Agricultural University, Wuhan 430070, China; wqi@webmail.hzau.edu.cn (Q.W.); zhuyanchun@webmail.hzau.edu.cn (Y.Z.); zouxiao@webmail.hzau.edu.cn (X.Z.); lifengfeng@mail.hzau.edu.cn (F.L.); zhangjialiang@webmail.hzau.edu.cn (J.Z.); kangziyi@webmail.hzau.edu.cn (Z.K.); lixuefei@webmail.hzau.edu.cn (X.L.); 2 National Key Laboratory of Crop Genetic Improvement, Wuhan 430070, China; yongjunlin@mail.hzau.edu.cn; 3 College of Life Science and Technology, Huazhong Agricultural University, Wuhan 430070, China

**Keywords:** cell elongation, cell proliferation, cytokinin, nitrogen deficiency, rice, root meristem, seminal root growth

## Abstract

Rice (*Oryza sativa* L.) seedlings grown under nitrogen (N) deficiency conditions show a foraging response characterized by increased root length. However, the mechanism underlying this developmental plasticity is still poorly understood. In this study, the mechanism by which N deficiency influences rice seminal root growth was investigated. The results demonstrated that compared with the control (1 mM N) treatment, N deficiency treatments strongly promoted seminal root growth. However, the N deficiency-induced growth was negated by the application of zeatin, which is a type of cytokinin (CK). Moreover, the promotion of rice seminal root growth was correlated with a decrease in CK content, which was due to the N deficiency-mediated inhibition of CK biosynthesis through the down-regulation of CK biosynthesis genes and an enhancement of CK degradation through the up-regulation of CK degradation genes. In addition, the N deficiency-induced decrease in CK content not only enhanced the root meristem cell proliferation rate by increasing the meristem cell number via the down-regulation of *OsIAA3* and up-regulation of root-expressed *OsPLTs*, but also promoted root cell elongation by up-regulating cell elongation-related genes, including root-specific *OsXTHs* and *OsEXPs*. Taken together, our data suggest that an N deficiency-induced decrease in CK content promotes the seminal root growth of rice seedlings by promoting root meristem cell proliferation and cell elongation.

## 1. Introduction

Rice (*Oryza sativa* L.) is one of the most important food crops in the world [1]. In the past 50 years, rice yield has steadily increased worldwide, partly owing to an increase in nitrogen (N) application. However, at present, the average recovery efficiency of N fertilizer (the percentage of fertilizer N recovered in aboveground plant biomass at the end of the cropping season) is only 33% at the field level [2]. High N input and low N use efficiency not only increase crop production costs but also cause severe environmental pollution [3,4]. Therefore, decreasing N application is an important goal of sustainable agriculture. However, decreasing N application may lead to N deficiency and affect rice root growth, and the underlying mechanism by which N deficiency affects rice root growth is still poorly understood.

Studies of crop responses to N deficiency have focused on the root [5,6], which is the plant organ that is most important for acquiring soil nutrients [7,8]. The developmental plasticity of root architecture is crucial for the acclimation of crops to unfavorable environments, including those that induce N stress. For example, a steeper and deeper root system more efficiently absorbs N in deep soil layers [9]. Root growth is influenced by several external and internal factors, including N availability and phytohormone homeostasis [10,11,12,13]. In general, a supraoptimal N supply inhibits root growth, and the decrease in root size can lead to decreased N uptake [14,15,16,17]. In contrast, N deficiency promotes root growth, and the increase in root size can improve N uptake ability [9,18]. Similarly, supraoptimal levels of the phytohormone cytokinin (CK) inhibit root growth [19], whereas a mild decrease in CK content promotes root growth [19,20,21]. These findings provide evidence that both N and CK are involved in mediating root growth.

CK regulates root growth in a dose-dependent manner [22]. We previously found that a threshold CK content is required for the rapid growth of rice seminal roots, but that supraoptimal CK levels inhibit growth [19]. Usually, the CK contents in roots cultured with high or moderate concentrations of N are supraoptimal for root growth, and thus a mild decrease in CK content promotes root growth. For example, a mild decrease in CK content achieved through overexpression of the CK degradation gene *CYTOKININ OXIDASE DEHYDROGENASE* (*CKX*) or mutation of the CK biosynthesis gene *ISOPENTENYLTRANSFERASE* (*IPT*) can promote primary root growth in *Arabidopsis* grown under moderate concentrations of N [20,21]. In contrast, without N application, the endogenous CK content in rice seedlings is optimal for growth of the seminal roots, and thus either a decrease or an increase in CK content leads to growth inhibition of the seminal root [19]. In addition, it has been reported that N treatment can increase CK content in roots [23]. These results suggest that N concentration is closely associated with CK content in the root. However, the mechanism by which the interaction between N and CK mediates rice root growth remains elusive.

Root growth is mainly determined by root meristem cell proliferation and root cell elongation [24,25,26]. The meristem cell proliferation rate is positively correlated with meristem cell number and meristem cell division activity [26]. The root meristem cell number is antagonistically regulated by many regulators, including PLETHORA (PLT) and SHORT HYPOCOTYL2/INDOLE-3-ACETIC ACID3 (SHY2/IAA3) [26,27]; and the meristem cell division activity is positively correlated with the transcription level of cyclin and cyclin-dependent protein kinase genes, such as *CycB1;1* and *CDKB2;1* [24,28]. *PLT* genes encode APETALA2 (AP2) transcription factors and are essential for root meristem maintenance [27]. In *Arabidopsis*, *plt1plt2* double mutants show a severe reduction in root meristem cell number, while the ectopic overexpression of *PLT2* leads to an increased number of meristematic cells and increased meristem size [27,29]. SHY2/IAA3 controls the root meristem cell number by promoting the mitotic-to-endocycle transition in the root, which in turn decreases the meristematic cell number and reduces the root meristem size [19,26]. Plants with a loss-of-function mutation in *SHY2/IAA3* have a larger-than-usual meristem, whereas those with a gain-of-function mutation in *SHY2/IAA3* have a smaller meristem than the wild type [25,30]. XYLOGLUCAN ENDOTRANSGLUCOSYLASE/HYDROLASE (XTH) and EXPANSIN (EXP) proteins play important roles in mediating root cell elongation [31,32], and thus mutations in *EXP* or *XTH* genes have been found to result in short root cells and short roots. For example, the *Arabidopsis atxth31* loss-of-function mutant has shorter root cells and shorter roots than the wild type [33], and the lengths of roots and root cells in *OsEXPB2* RNA interference lines were significantly shorter than those in wild-type rice plants [34]. In rice, the transcription levels of *OsXTH* and *OsEXP* genes, such as *OsXTH1*, *OsEXP3*, and *OsEXPB4*, which are specifically expressed in the root, are closely correlated with the lengths of mature cells in rice seminal root [19].

CK has been shown to regulate the expression of the genes regulating root meristem cell number and root cell elongation [27,35]. Previous studies have reported that some hormone response elements, including the CK-response element (AGATT), exist in promoter regions of the root-expressed *OsPLTs* [35,36]. *OsPLT1–6* can strongly respond to CK because there are more than seven AGATT elements in each promoter region [35]. In addition, it has been reported that CK can up-regulate *OsIAA3* and down-regulate *OsXTHs* and *OsEXPs* [19]. However, up to now, it has been unclear whether N and CK collaboratively regulate root meristem cell number and cell elongation by modulating transcription levels of *OsPLTs*, *OsIAA3*, *OsXTHs*, and *OsEXPs*.

Rice seminal roots are the first roots to emerge from seeds after germination and are responsible for water uptake and nutrient absorption during seedling establishment [37]. In this study, the influence of N deficiency on CK metabolism in rice seminal roots was investigated. Furthermore, the mechanism by which N and CK interact to regulate rice seminal root growth was clarified. We provide evidence that N deficiency reduces CK content by inhibiting CK biosynthesis and promoting CK degradation, which in turn promotes rice seminal root growth. Specifically, the decrease in CK content results in the down-regulation of *OsIAA3* and up-regulation of *OsPLT* genes, leading to an increase in root meristem cell number and cell proliferation rate, and an up-regulation of the *OsXTH* and *OsEXP* genes, which promote cell elongation.

## 2. Materials and Methods

### 2.1. Plant Material and Growth Conditions

Japonica rice Nipponbare (*Oryza sativa* L.) was used in this study. Rice seeds were sterilized, soaked, and germinated according to the method of Zou et al. [38]. The germinated rice seeds were grown in an artificial climate incubator (HP 1500 GS, Ruihua Instrument & Equipment Co., Ltd., Wuhan, China) with a 12-h light (29 °C)/12-h dark (26 °C) photoperiod.

### 2.2. Chemicals and Treatments

Zeatin (Z) (Sigma-Aldrich Trading Co., Ltd., Shanghai, China), a mixture of *trans*-Z (*t*Z) and *cis*-Z (*c*Z), was dissolved in dimethyl sulfoxide (DMSO) and diluted to the desired concentration with distilled water. NH_4_NO_3_ (Shanghai Yuanye Biotechnology Co., Ltd., Shanghai, China), which was used as the source of N, was dissolved directly in distilled water and diluted to the desired concentration. The solution for the control treatment contained 0.5 mM NH_4_NO_3_ (1 mM N, 1 N), the solutions for the N deficiency treatments contained 0.125 mM NH_4_NO_3_ (0.25 mM N, 1/4 N), 0.03125 mM NH_4_NO_3_ (0.0625 mM N, 1/16 N), or 0 mM NH_4_NO_3_ (0 mM N, 0 N), and the solution for the N deficiency + Z treatment contained 0 mM N and 4 nM Z (0 N + Z). All solutions also contained 0.3 mM NaH_2_PO_4_, 0.15 mM K_2_SO_4_, 0.15 mM CaCl_2_, 0.3 mM MgCl_2_, 0.08 mM Na_2_SiO_3_, 4.6 μM MnSO_4_, 0.05 μM Na_2_MoO_4_, 24.5 μM H_3_BO_3_, 0.35 μM ZnSO_4_, 0.15 μM CuSO_4_, 32.7 μM Fe (II)-EDTA (Ethylene Diamine Tetraacetic Acid), and 70.8 μM C_6_H_7_O_8_. The pH of each solution was adjusted to 6.5 with 0.1 N HCl or NaOH. Since the Z solutions also contained DMSO, DMSO was added to the solutions without Z to a final concentration of 0.01% (*v*/*v*). The germinated rice seeds were incubated in different solutions for one day, two days, three days, or four days. All solutions were refreshed every two days.

### 2.3. CK Content Analysis

The germinated seeds were incubated in a solution containing 0 N, 1/16 N, 1/4 N, or 1 N (control). After four days of treatment, the seminal roots of the rice seedlings were collected and frozen at −80 °C until use. CKs were extracted, purified, and quantified according to previously described methods [19,39]. Data are reported as means ± standard deviation (SD) of three independent biological replicates.

### 2.4. RNA Isolation and Quantitative Real-Time PCR (qRT-PCR) Analysis

The germinated seeds were incubated in a solution containing 0 N, 0 N + Z, or 1 N as a control for one day, two days, or four days; then, the seminal roots of the rice seedlings were collected and frozen at −80 °C until use. Total RNA was extracted according to Liu et al. [40]. First-strand cDNA was synthesized using the Fastking RT Kit (Tiangen Biotechnology Co., Ltd., Beijing, China). The relative expression level was analyzed by qRT-PCR according to the method described previously [40]; the gene-specific primers are listed in Supplementary Table S1. The relative expression level was calculated using the comparative threshold method, using *OsACTIN* as an internal control. Three biological replicates with three technical replicates were used for the statistical analyses and error range analyses, and data are presented as the means ± SD. The relative expression levels of CK metabolism genes, root meristem size-related genes, and root cell elongation-related genes in rice seminal root are presented in Supplementary Figure S1.

### 2.5. Quantitative Analysis of Root Phenotypes

The seminal roots of the rice seedlings were selected for the quantitative analysis of root phenotypes. Seminal root tips were stained with 4’,6-diamino-2-phenylhydrazine solution to measure root meristem size and meristem cell number, and mature cells in rice seminal roots were stained with propidium iodide solution to measure mature cell length. The staining methods have been reported previously [19]. Images were taken on a confocal microscope (Leica SP8, Leica Corporation, Solms, Germany). Root length was measured with Image J software 1.46 (National Institutes of Health, Bethesda, MD, USA). Root meristem size was determined by measuring the length from the quiescent center to the first elongated epidermal cell. Root meristem cell number was expressed as the number of cortex cells from the quiescent center to the first elongated cell. Root growth rate was calculated as described previously [41]. Cell proliferation rate was calculated using the following equation: cell proliferation rate = root growth rate/mature cell length. Data are presented as means ± SD calculated from 10 biological replicates.

### 2.6. Statistical Analysis

Statistical analysis was performed using an independent samples *t*-test or a one-way analysis of variance followed by Duncan’s multiple range test, with at least three biological replicates for each treatment. Values of *p* < 0.05 were considered statistically significant. All data are presented as means ± SD.

### 2.7. Accession Numbers

Sequence data from this study can be found in the Chinese national rice data center (http://www.ricedata.cn/gene/index.htm) under the following accession numbers: *OsIPT1* (Os03g0358900), *OsIPT3* (Os05g0311801), *OsIPT4* (Os03g0810100), *OsIPT5* (Os07g0211700), *OsIPT7* (Os05g0551700), *OsIPT8* (Os01g0688300), *OsCKX1* (Os01g0187600), *OsCKX2* (Os01g0197700), *OsCKX3* (Os10g0483500), *OsCKX4* (Os01g0940000), *OsCKX5* (Os01g0775400), *OsCKX6* (Os02g0220000), *OsCKX7* (Os06g0572300), *OsCKX8* (Os04g0523500), *OsCKX9* (Os05g0374200), *OsCKX10* (Os06g0572300), *OsCKX11* (Os08g0460600), *OsPLT1* (Os04g0653600), *OsPLT2* (Os06g0657500), *OsPLT3* (Os02g0614300), *OsPLT4* (Os04g0504500), *OsPLT5* (Os01g0899800), *OsPLT6* (Os11g0295900), *OsCycB1;1* (Os01g0805600), *OsCDKB2;1* (Os08g0512600), *OsIAA3* (Os01g0231000), *OsXTH1* (Os07g0529700), *OsXTH2* (Os11g0539200), *OsXTH4* (Os08g0240500), *OsXTH13* (Os02g0280200), *OsXTH15* (Os06g0336001), *OsXTH16* (Os04g0604900), *OsXTH25* (Os10g0577500), *OsEXP3* (Os05g0276500), *OsEXP11* (Os01g0274500), *OsEXP12* (Os03g0155300), *OsEXP13* (Os02g0267200), *OsEXP15* (Os03g0155700), *OsEXP17* (Os06g0108600), *OsEXPB2* (Os10g0555700), *OsEXPB8* (Os03g0102500), and *OsACTIN* (Os03g0718100).

## 3. Results

### 3.1. N Deficiency-Induced Growth of Rice Seminal Roots was Negated by Application of CK

**Figure 1:**
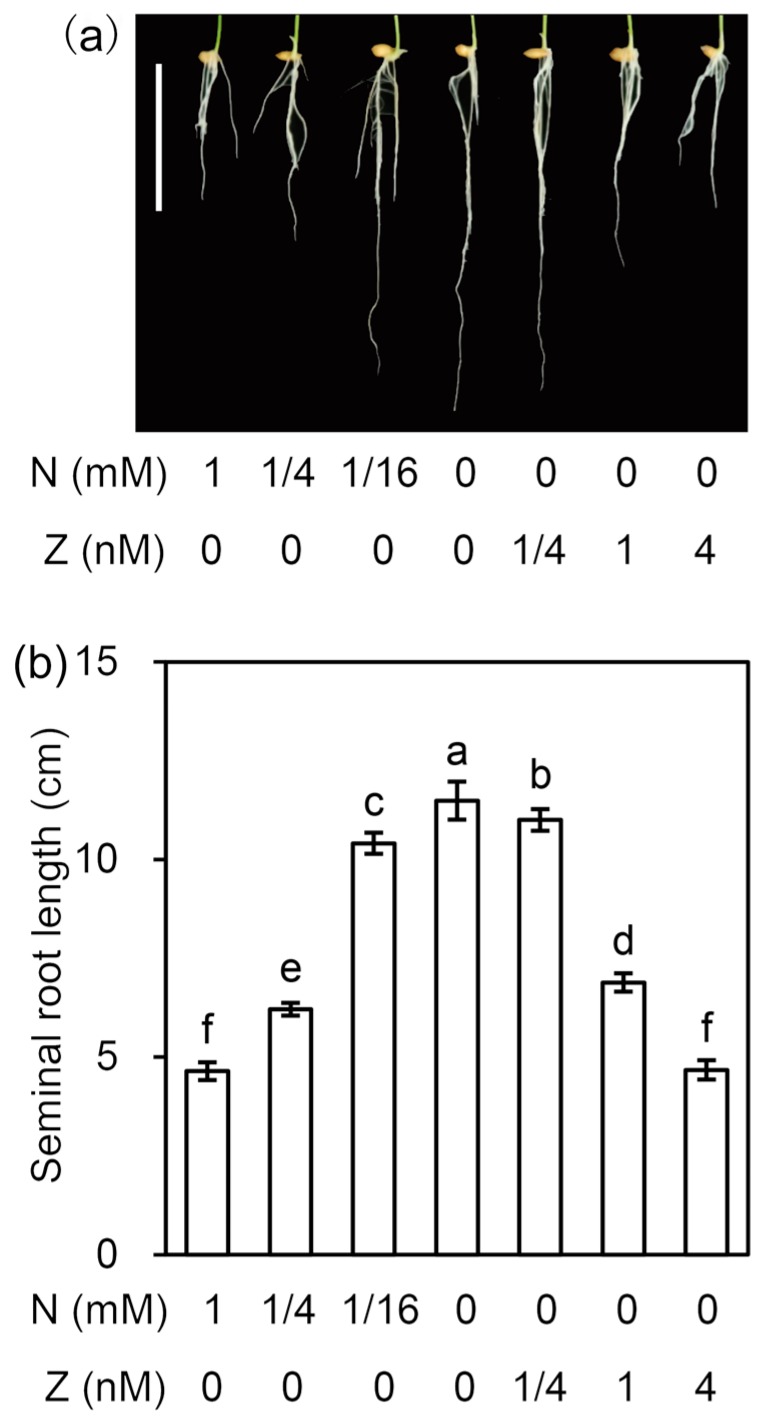
Effects of the N and Z treatments on rice seedling seminal root growth. (**a**) Phenotypic comparison of seminal roots of rice seedlings grown under different treatments. Scale bar is 5 cm. (**b**) Seminal root lengths under different treatments. In this experiment, germinated rice seeds were incubated in different solutions for four days, then photographs were taken, and the lengths of the seminal roots were measured. The data are the means ± SD calculated from 10 biological replicates. Significant differences (*p* < 0.05) are indicated by different letters (a, b, c, d, e, f). N, nitrogen; Z, zeatin.

In order to investigate the effect of N deficiency on rice seminal root growth, rice seedlings were cultivated hydroponically under control (1 N) and N deficiency (1/4 N, 1/16 N, and 0 N) conditions. After four days of cultivation, the average lengths of rice seminal roots treated with 1 N, 1/4 N, 1/16 N, and 0 N were 4.6 cm, 6.2 cm, 10.4 cm, and 11.5 cm, respectively (Figure 1). The 1/4 N, 1/16 N, and 0 N treatments increased the rice seminal root lengths by 35%, 126%, and 150%, respectively, compared with those of the control. This result demonstrated that N deficiency promoted rice seminal root growth, and that the decrease in N was accompanied by an increase in rice seminal root length. Furthermore, the application of Z (a type of CK) at concentrations of 0.25–4 nM significantly inhibited the N deficiency-induced growth of rice seminal roots, and 4 nM Z completely blocked the N deficiency-induced growth (Figure 1). This result suggested that N deficiency might promote rice seminal root growth by controlling the CK content.

### 3.2. N Deficiency Reduced CK Content in Rice Seminal Roots

To test whether N deficiency induces rice seminal root growth by controlling the content of endogenous CKs, the effects of N deficiency on the contents of endogenous Z, dihydrozeatin (DZ), and isopentenyladenine (iP), which are the predominant CKs in higher plants [42], were analyzed. Since the previous results clearly demonstrated comparable activities of *trans*-Z (*t*Z) and *cis*-Z (*c*Z) in the inhibition of rice seminal root elongation [43], thus the total content of Z, including *t*Z and *c*Z, was analyzed in this study. As shown in Table 1, the content of Z was significantly higher than that of DZ and iP, indicating that Z is the predominant CK in rice seminal roots. Under control (1 N) treatment, the content of Z in the seminal root was relatively high (198.4 ± 11.1 pmol·g^−1^ FW) (Table 1). By contrast, under the N deficiency treatments, the content of Z in seminal roots decreased significantly; the contents of Z under the 1/4 N, 1/16 N, and 0 N treatments were only 52%, 36%, and 34% of that of the control, respectively. However, neither DZ nor iP content was affected by N deficiency treatments compared with control (1 N) treatment (Table 1). This result indicated that N deficiency reduced CK content by reducing Z content rather than by controlling DZ or iP content in rice seminal roots.

### 3.3. N Deficiency Inhibited CK Biosynthesis and Promoted CK Degradation by Affecting the Transcription Levels of CK Metabolic Genes in Rice Seminal Roots

**Figure 2:**
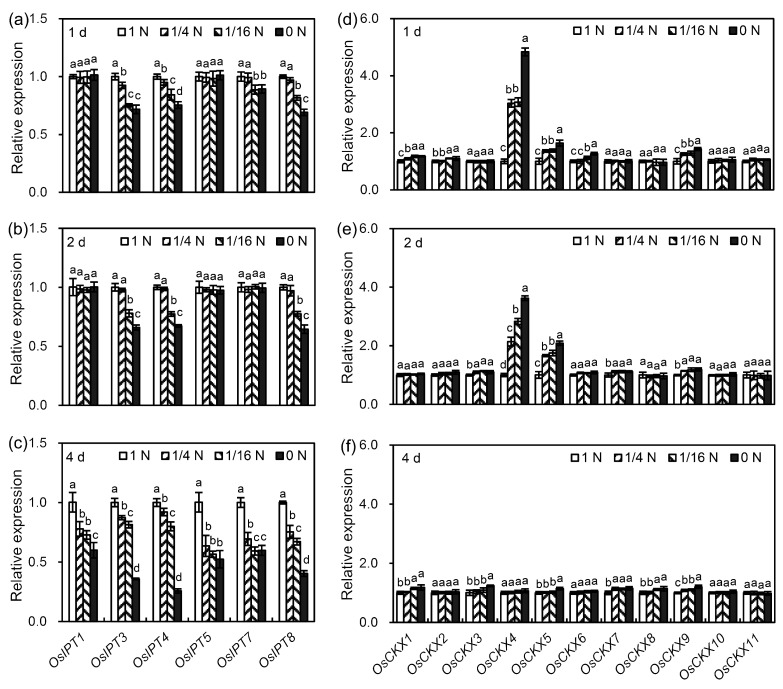
Comparison of the relative expression levels of CK metabolic genes between control and N deficiency treatments using qRT-PCR. (**a**–**c**) Relative expression levels of CK biosynthesis genes after one day, two days, and four days of treatment. (**d**–**f**) Relative expression levels of CK degradation genes after one day, two days, and four days of treatment. Germinated rice seeds were incubated in control (1 N) or N deficiency (1/4 N, 1/16 N, and 0 N) solution. After one day, two days, and four days of treatment, the seminal roots of rice seedlings were sampled for qRT-PCR analyses. Data are presented as the means ± SD. Three biological replicates with three technical replicates were used for the statistical and error range analyses. The relative expression levels of CK metabolic genes in the control were set as 1. Significant differences (*p* < 0.05) in the relative expression level of the same gene between control and N deficiency treatments are indicated by different letters (a, b, c, d). N, nitrogen; 1 N, 1 mM N; 1/4 N, 1/4 mM N; 1/16 N, 1/16 mM N; 0 N, 0 mM N.

CK homeostasis is mainly controlled by CK biosynthesis and degradation [44,45]. In rice, six *OsIPTs* (*OsIPT1*, *OsIPT3*, *OsIPT4*, *OsIPT5*, *OsIPT7*, and *OsIPT8*) and 11 *OsCKXs* (*OsCKX1–11*) are the key genes modulating CK biosynthesis and degradation, respectively [46,47]. Thus, to investigate the mechanism by which N deficiency regulates CK content, the effects of N deficiency on the transcription levels of these six *OsIPTs* and 11 *OsCKXs* in rice seminal roots were investigated. On the one hand, N deficiency could reduce CK content by decreasing CK biosynthesis via the down-regulation of *OsIPT* genes. As shown in Figure 2a, after one day of treatment, of the six *OsIPT* genes tested, there were significant differences in the transcription levels between control and N deficiency treatments for four genes; the transcription levels of *OsIPT3* and *OsIPT4* were significantly down-regulated in response to all the N deficiency treatments, and the transcription levels of *OsIPT7* and *OsIPT8* were significantly down-regulated in response to the 1/16 N and 0 N treatments. After two days of treatment, the transcription levels of *OsIPT3*, *OsIPT4*, and *OsIPT8* were significantly down-regulated by the 1/16 N and 0 N treatments (Figure 2b). After four days of treatment, the transcription levels of all six *OsIPT* genes were significantly down-regulated by treatment with ≤1/4 N (Figure 2c). These results suggest that N deficiency might reduce CK content by inhibiting CK biosynthesis via the down-regulation of *OsIPT* genes. On the other hand, N deficiency could reduce CK content by promoting CK degradation via the up-regulation of *OsCKX* genes. As shown in Figure 2d–f, *OsCKX10* and *OsCKX11* did not respond to N deficiency treatments, but the other eight *OsCKX* genes were up-regulated to different extents by different N deficiency treatments. Among the nine N deficiency-responsive *OsCKX* genes, *OsCKX4* responded most strongly to N deficiency; the transcription level of *OsCKX4* was up-regulated by more than 2-fold after one and two days of N deficiency treatment.

### 3.4. N Deficiency-Induced Decrease in CK Content Enhanced Root Meristem Cell Proliferation Rate and Promoted Root Cell Elongation

**Figure 3:**
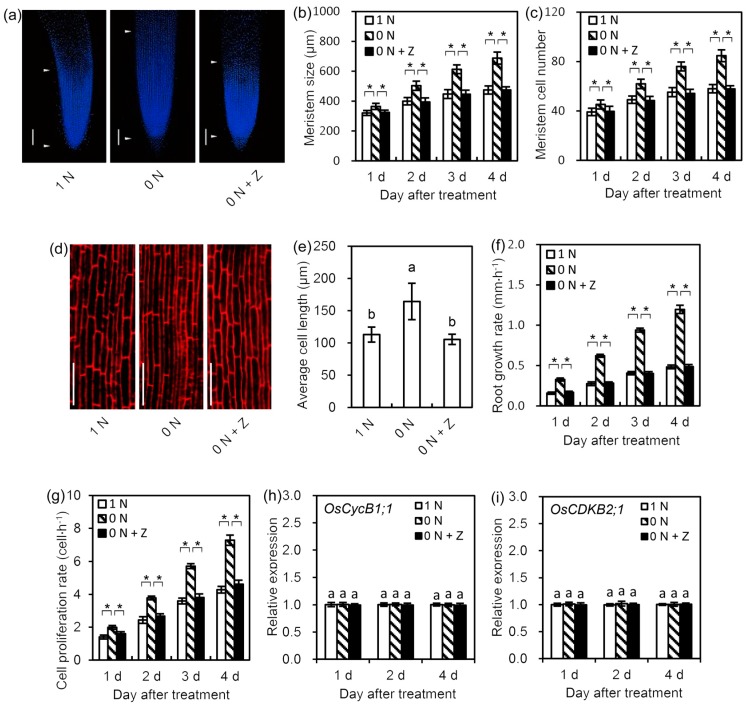
Effects of 1 N, 0 N, and 0 N + Z treatments on root phenotype, root growth rate, and meristem cell proliferation rate in rice seminal roots. Germinated rice seeds were incubated with a solution containing 0 N, 0 N + Z, or 1 N as a control for one day, two days, three days, or four days. (**a**–**c**) Comparison of root phenotype, meristem size, and meristem cell number between different treatments in rice seminal roots. The meristems of the rice seminal roots were visualized using 4′,6-diamidino-2-phenylindole staining and measured. Root meristem size was determined by measuring the length from the quiescent center to the first elongated epidermal cell. Meristem cell number in rice seminal root was expressed as the number of cortex cells from the quiescent center to the first elongated cell. (**d**,**e**) Comparison of mature cell phenotype and mature cell length between different treatments in rice seminal roots. The lengths of cells in the mature zones of the seminal roots were visualized using propidium iodide staining and measured. (**f**,**g**) Comparison of root growth rate and root cell proliferation rate between different treatments in rice seminal roots. Cell proliferation rate = root growth rate / mature cell length. (**h**,**i**) Comparison of the relative expression levels of root meristem cell division-related genes between different treatments in rice seminal roots. The relative expression levels of *OsCycB1;1* and *OsCDKB2;1* were used to indicate the meristem cell division activities in rice seminal roots. Bars = 100 μm for (**a**,**d**). Data are presented as means ± SD calculated from 10 biological replicates, and significant differences (*p* < 0.05) are indicated by asterisk or different letters (a, b). N, nitrogen; 1 N, 1 mM N; 0 N, 0 mM N; 0 N + Z, 0 mM N + 4 nM Z; Z, zeatin.

We investigated the effects of control (1 N), N deficiency (0 N), and N deficiency + Z (0 N + Z) treatments on seminal root meristem cell proliferation rate and mature cell length to clarify the mechanism by which the N deficiency-induced decrease in CK content increases seminal root length. The results demonstrated that the meristem sizes and the cell numbers of the seminal roots in the N deficiency treatment group increased significantly compared with those in the control group, but this effect was completely negated by the application of Z (Figure 3a–c). Moreover, the lengths of mature cells in the mature zone of rice seminal roots were also significantly increased by N deficiency treatment; the average root cell length in the mature zone was increased by 45.5% under N deficiency treatment (Figure 3d,e). However, this increase in cell length was completely negated by the addition of Z (Figure 3d,e). Further, the results demonstrate that N deficiency strongly enhanced the root growth rate and root meristem cell proliferation rate, but these effects were negated by the addition of Z (Figure 3f,g). In addition, compared with control treatment, 0 N and 0 N + Z treatments had no influence on the transcription level of root meristem cell division-related genes, such as *OsCycB1;1* and *OsCDKB2;1* (Figure 3h,i). These results suggest that the N deficiency-induced decrease in CK content enhanced the root meristem cell proliferation rate and promoted root cell elongation, and that the enhanced cell proliferation rate was contributed by the increased meristem cell number rather than by the changed meristem cell division activity.

### 3.5. Decrease in CK Content Increased Root Meristem Cell Number by Affecting the Transcription Levels of Root Meristem Size-Related Genes

**Figure 4:**
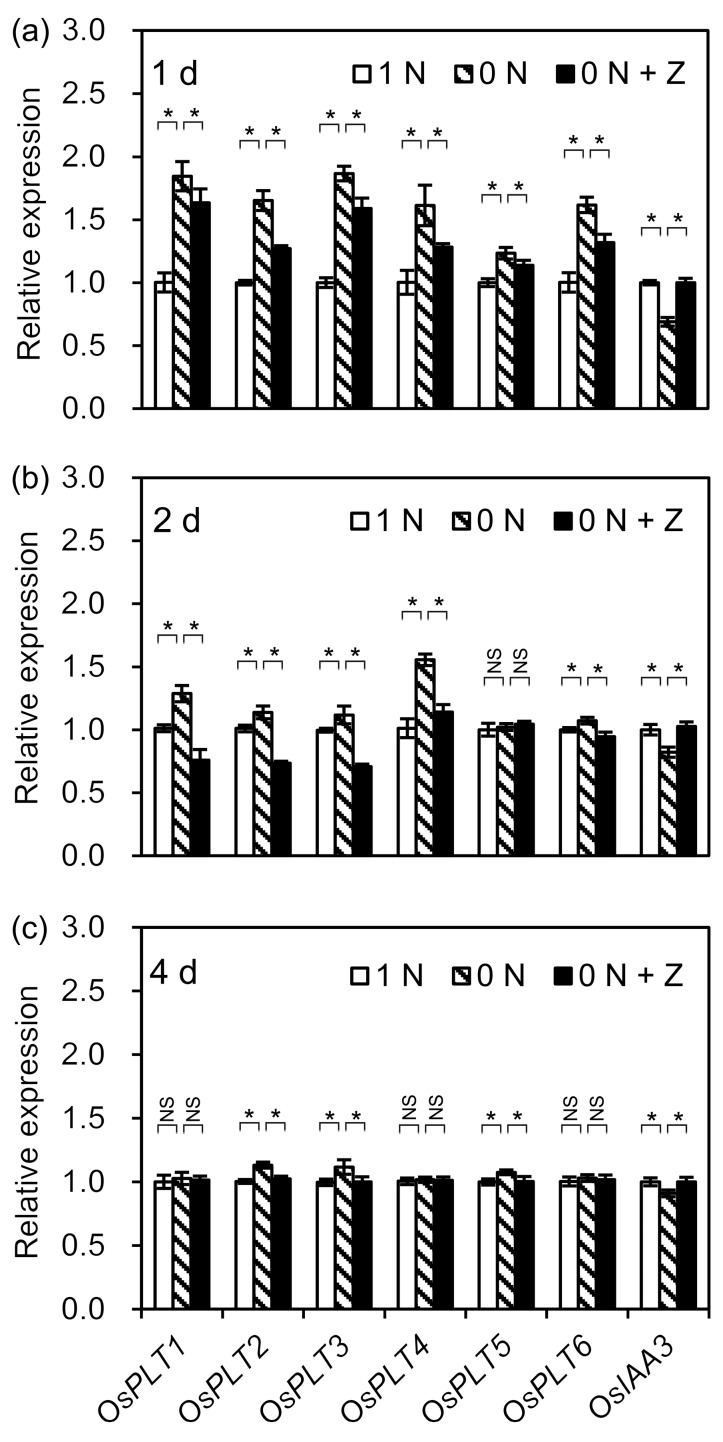
Transcriptional regulation of root meristem size-related genes in rice seminal roots under control, N deficiency, and N deficiency + Z treatments. Germinated rice seeds were incubated in a solution containing 0 N, 0 N + Z, or 1 N as a control. Rice seminal roots were collected after one day (**a**), two days (**b**), and four days (**c**) of incubation, and qRT-PCR was used to compare the transcription levels of root meristem size-related genes, including six *OsPLTs* and *OsIAA3*, under each treatment at each time point. Data are presented as means ± SD. Three biological replicates with three technical replicates each were included in statistical and error range analyses. The relative expression levels of root meristem size-related genes in control roots were set as 1. Asterisk indicates a significant difference (*p* < 0.05) in the relative expression level of the same gene in different treatments. N, nitrogen; NS, not significant; 1 N, 1 mM N; 0 N, 0 mM N; 0 N + Z, 0 mM N + 4 nM Z; Z, zeatin.

It has been reported that root meristem size is positively regulated by PLT and negatively regulated by IAA3 [25,27]. There are 10 *OsPLT* genes in rice; among them, only six (*OsPLT1–6*) are expressed in roots [35]. Therefore, to clarify whether the N deficiency-induced decrease in CK increased meristem cell number by affecting the transcription of meristem size-related genes in rice seminal roots, the effects of control (1 N), N deficiency (0 N), and N deficiency + Z (0 N + Z) treatments on the transcription levels of *OsPLT1–6* and *OsIAA3* were investigated. After one day of N deficiency treatment, the transcription levels of *OsPLT1–6* were significantly increased, while the transcription level of *OsIAA3* was significantly decreased, but these effects were antagonized by the application of Z (Figure 4a). After two days of treatment, the transcription levels of most of the root meristem size-related genes changed in response to N deficiency, but these transcriptional changes were smaller in the presence of Z (Figure 4b). After four days of treatment, the transcription levels of the four meristem size-related genes under N deficiency were still slightly but significantly different from those under the control condition, and the transcriptional regulation of these genes was also antagonized by the application of Z (Figure 4c). These results suggest that the N deficiency-induced decrease in CK content led to an increased root meristem cell number through the down-regulation of *OsIAA3* and the up-regulation of root-expressed *OsPLTs*.

### 3.6. Decrease in CK Content Increased Root Cell Length by Up-Regulating Transcription of Root Cell Elongation-Related Genes

**Figure 5:**
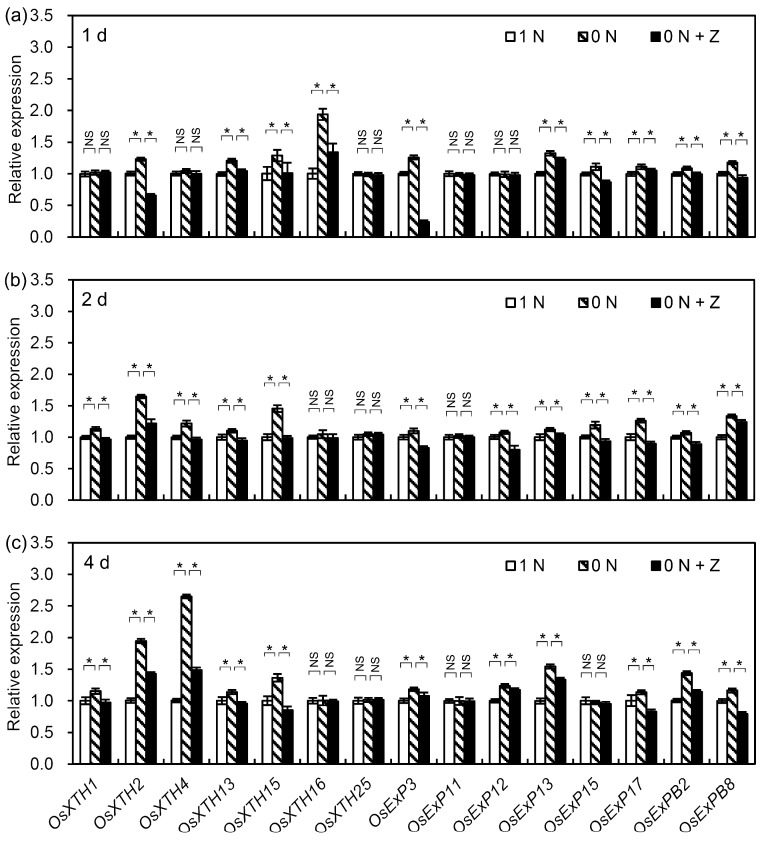
Transcriptional regulation of cell elongation-related genes in rice seminal roots under control, N deficiency, and N deficiency + Z treatments. Germinated rice seeds were incubated in a solution containing 0 N, 0 N + Z, or 1 N as a control. Rice seminal roots were collected after one day (**a**), two days (**b**), and four days (**c**) of incubation, and qRT-PCR was used to compare the transcription levels of root cell elongation-related genes, including seven *OsXTHs* and eight *OsEXPs*, under each treatment at each time point. Data are presented as means ± SD. Three biological replicates with three technical replicates each were included in statistical and error range analyses. The relative expression levels of root cell elongation-related genes in control roots were set as 1. Asterisk indicates a significant difference (*p* < 0.05) in the relative expression level of the same gene in different treatments. N, nitrogen; NS, not significant; 1 N, 1 mM N; 0 N, 0 mM N; 0 N + Z, 0 mM N + 4 nM Z; Z, zeatin.

It has been reported that seven *OsXTHs* and eight *OsEXPs* are specifically expressed in rice roots [31,32], and that these root-specific genes play important roles in regulating root cell elongation in rice seedlings [31,32]. To clarify whether the N deficiency-induced decrease in CK increased the cell length in rice seminal roots by affecting the transcription levels of cell elongation-related genes, the effects of the control (1 N), N deficiency (0 N), and N deficiency + Z (0 N + Z) treatments on the transcription levels of the root-specific *OsXTHs* (*OsXTH1*, *OsXTH2*, *OsXTH4*, *OsXTH13*, *OsXTH15*, *OsXTH16*, and *OsXTH25*) and *OsEXPs* (*OsEXP3*, *OsEXP11*, *OsEXP12*, *OsEXP13*, *OsEXP15*, *OsEXP17*, *OsEXPB2*, and *OsEXPB8*) were investigated. After one day of N deficiency treatment, two-thirds of the root-specific *OsXTHs* and *OsEXPs* were up-regulated (Figure 5a). After two days of treatment, all root-specific *OsXTHs* and *OsEXPs* except for *OsXTH16*, *OsXTH25*, and *OsEXP11* were up-regulated by the N deficiency treatment (Figure 5b). After four days of treatment, most of the root-specific *OsXTH* and *OsEXP* genes were still up-regulated under the N deficiency treatment (Figure 5c). At every time point, the effects on transcription were reduced in the presence of Z (Figure 5a–c). Taken together, all root-specific *OsXTHs* and *OsEXPs* except for *OsXTH25* and *OsEXP11* were up-regulated to different extents by N deficiency, and the up-regulation of these genes was inhibited by Z treatment. These results indicate that the N deficiency-induced decrease in CK content increased root cell length by up-regulating the transcription levels of cell elongation-related genes, including six *OsXTHs* and seven *OsEXPs*, in rice seminal roots.

## 4. Discussion

### 4.1. Increasing Root Length is a Strategy for Responding to N Deficiency

Rice seminal roots are the first roots to emerge from seeds after germination and are responsible for water uptake and nutrient absorption during seedling establishment [37]. Here, we provide evidence that N deficiency treatments strongly promote seminal root growth of rice seedlings under a relatively short duration (four days) of N deficiency. Moreover, even under a long duration (30 days or 60 days), rice seminal root growth is still promoted by N deficiency treatments (Supplementary Figure S2). This result is consistent with previous reports that N deficiency treatment promotes root growth in other plants, including wheat [48] and *Arabidopsis* [49,50]. Root length is an essential parameter allowing crops to increase N use efficiency, as more and deeper roots may improve the uptake of N from deeper soil layers and reduce N losses to the environment [9]. Taken together, these findings suggest that increasing root length is a strategy used by plants to respond to N deficiency. However, because N is an essential element, a long duration of severe N deficiency such as 0 N treatment inhibits adventitious root production and reduces the whole root system volume (Supplementary Figure S2).

### 4.2. N Deficiency Promotes Rice Seminal Root Growth by Reducing CK Content 

We found that the CK contents in rice seminal roots in the N deficiency treatment groups were lower than those of the control, and that a decrease in the concentration of N was accompanied by a decrease in the content of CKs as well as a longer seminal root (Figure 1, Table 1). Moreover, the induction of rice seminal root growth was significantly negated by CK application (Figure 1). Previous work has shown that the application of high or moderate concentrations of N induces CK accumulation in the roots of dicotyledonous plants such as *Arabidopsis* [51] and monocotyledonous crops, including rice [52], wheat [48], maize [53], and barley [54]; this accumulated CK inhibits root growth [19,55]. Usually, CK content in the roots of plants incubated with high or moderate concentrations of N is supraoptimal for rapid root growth, and thus mild decreases in CK content promote root growth [20,21]. All these findings lead to the conclusion that N deficiency promotes rice seminal root growth by reducing CK content. Moreover, our results indicated that N deficiency reduces CK content by reducing Z content rather than by controlling DZ or iP content in rice seminal roots (Table 1).

### 4.3. N Deficiency Reduces CK Content by Inhibiting CK Biosynthesis and Promoting CK Degradation

To investigate the regulatory mechanism by which N deficiency affects CK metabolism, the effects of N deficiency on the expression of six *OsIPTs* and 11 *OsCKXs* were analyzed in this study. We found that N deficiency inhibited CK biosynthesis and promoted CK degradation through down-regulation of the *OsIPT* genes and up-regulation of *OsCKX* genes (Figure 2a–f), respectively, which in turn led to reduced CK content. Our result is supported by studies in *Arabidopsis* and tobacco that found that expressing *CKX* genes specifically in the roots results in reduced CK content through the enhancement of root-specific degradation of CK [56]. Moreover, our result is also supported by experiments in rice showing that an increase in endogenous CK content achieved through the knockdown of *OsCKX4* or overexpression of *OsIPT* inhibits the growth of rice seminal roots [47,57]. In addition, our findings are also consistent with a study in rice in which *OsIPT4* was found to be responsible for the increased content of CK in roots in response to nitrite (a type of N fertilizer) treatment [23]. Taken together, these results suggest that N deficiency reduces CK content by inhibiting CK biosynthesis and promoting CK degradation.

### 4.4. N Deficiency-Induced Decrease in CK Content Promotes Rice Seminal Root Growth by Enhancing Meristem Cell Proliferation Rate through Increased Meristem Cell Number

The root meristem cell proliferation rate is positively correlated with meristem cell number and meristem cell division activity [26]. We found that the N deficiency-induced decrease in CK content increases the root meristem cell number by up-regulating *OsPLT1–6* and down-regulating *OsIAA3* (Figuers 3c,4), which in turn promotes rice seminal root growth by enhancing the root meristem cell proliferation rate (Figure 3g). However, the N deficiency-induced decrease in CK content could not affect meristem cell division activity in rice seminal root (Figure 3h,i). This result is consistent with a previous report that CK had no influence on meristem cell division activity in *Arabidopsis* primary root [24]. All the evidence suggests that the N deficiency-induced decrease in CK content increases root meristem cell number by up-regulating *OsPLT1–6* and down-regulating *OsIAA3*, which in turn promotes rice seminal root growth by enhancing the root meristem cell proliferation rate through an increased meristem cell number. Our result is supported by previous studies showing that a mild decrease in endogenous CK content promotes root growth [20,21], while CK treatment strongly inhibits root growth [19]. Our result is also consistent with the finding in rice that the transcription of all *OsPLT1–6* genes declined to the lowest level after 24 h of CK treatment [35], and that the transcription of the *OsIAA3* gene increased significantly within four days of CK treatment [19].

### 4.5. Decrease in CK Content Promotes Rice Seminal Root Growth by Promoting Root Cell Elongation

Root-specific *OsXTHs* and *OsEXPs* have been implicated in the control of rice root cell elongation [19,34]. Here, we provide evidence that all root-specific *OsXTHs* and *OsEXPs* except for *OsXTH25* and *OsEXP11* were up-regulated by N deficiency, but that this up-regulation was blocked by the application of Z (Figure 5a–c). This finding is consistent with our previous finding that the application of exogenous kinetin (a synthetic CK) down-regulated the transcription of *OsXTHs* and *OsEXPs*, such as *OsXTH1* and *OsEXP3*, which in turn inhibited rice seminal root growth [19]. These pieces of evidence suggest that the N deficiency-induced decrease in CK content promotes rice seminal root growth by promoting root cell elongation through the up-regulation of root-specific *OsXTHs* and *OsEXPs*.

## 5. Conclusions

**Figure 6:**
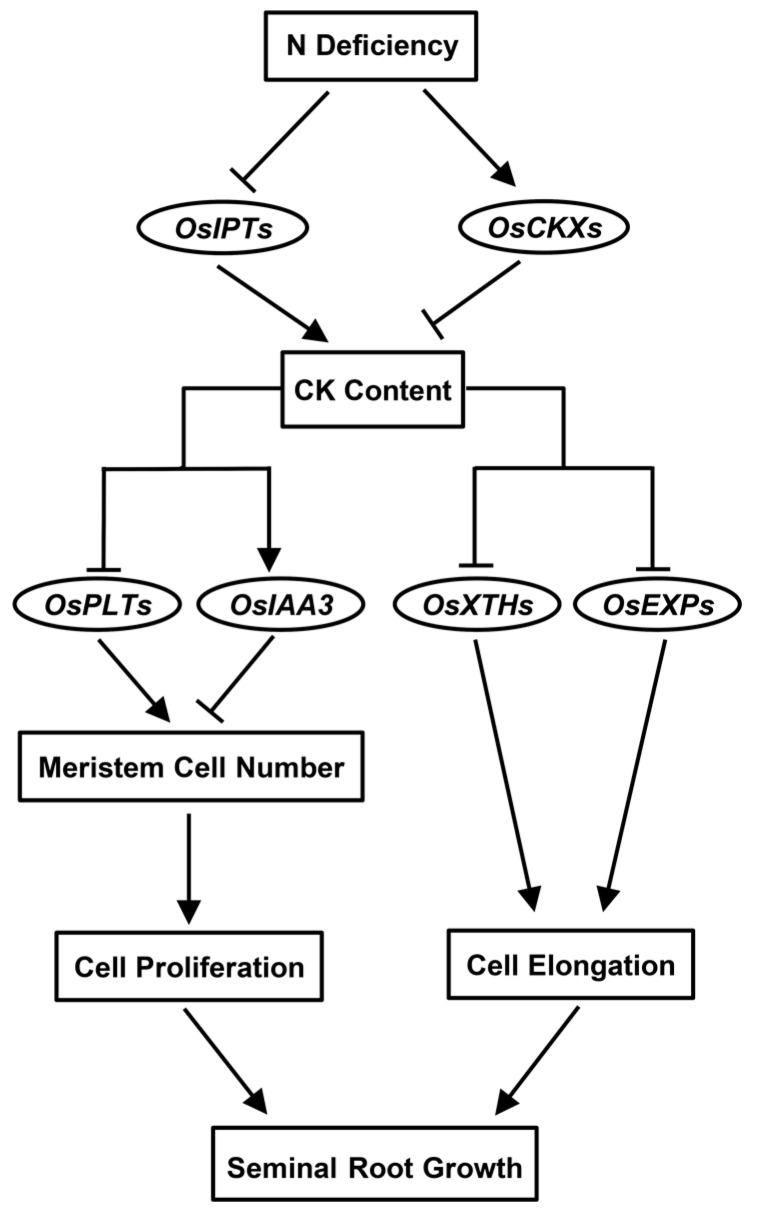
Suggested model for the regulation of seminal root responses to N deficiency in rice seedlings. N, nitrogen; CK, cytokinin.

In summary, our results demonstrate that N deficiency reduces CK content by inhibiting CK biosynthesis and promoting CK degradation through the down-regulation of CK biosynthesis genes and up-regulation of CK degradation genes, respectively (Figure 6). Moreover, our results indicate that the N deficiency-induced decrease in CK content promotes rice seminal root growth not only by enhancing the root meristem cell proliferation rate through an increased root meristem cell number, but also by promoting root cell elongation through the up-regulation of root-specific *OsXTHs* and *OsEXPs* (Figure 6). In addition, our results suggest that the N deficiency-induced decrease in CK content increases the root meristem cell number by up-regulating root-expressed *OsPLTs* and down-regulating *OsIAA3* (Figure 6). Taken together, our data suggest that an N deficiency-induced decrease in CK content promotes rice seminal root growth by promoting root meristem cell proliferation and root cell elongation. Further study is required to characterize the molecular mechanisms involved in the N-regulated transcription of *OsIPTs* and *OsCKXs*. Another important issue requiring further in-depth study is the role played by CK signaling in regulating the transcription of *OsIAA3*, *OsPLTs*, *OsXTHs*, and *OsEXP*s.

## Figures and Tables

**Table 1:** Contents of CKs in rice seminal roots under different N treatments. Germinated rice seeds were cultured in solutions containing different concentrations of N for four days, and then the contents of the CKs, including Z, DZ, and iP in rice seminal roots, were measured. Data are presented as means ± SD of three independent biological replicates. Significant differences (*p* < 0.05) in the content of the same type of CK between different treatments are indicated by different letters (a, b, c, d). CKs, cytokinins; DZ, dihydrozeatin; FW, fresh weight; iP, isopentenyladenine; N, nitrogen; 1 N, 1 mM N; 1/4 N, 1/4 mM N; 1/16 N, 1/16 mM N; 0 N, 0 mM N. Z, zeatin.

Treatments	Z (pmol g^−1^ FW)	DZ (pmol·g^−1^ FW)	iP (pmol·g^−1^ FW)
1 N	198.4 ± 11.1 a	1.12 ± 0.06 a	6.36 ± 0.16 a
1/4 N	102.3 ± 2.6 b	1.09 ± 0.07 a	6.32 ± 0.21 a
1/16 N	71.3 ± 2.8 c	1.13 ± 0.06 a	6.40 ± 0.17 a
0 N	68.2 ± 0.8 d	1.09 ± 0.04 a	6.30 ± 0.33 a

## Supplementary Materials

The following are available online at https://www.mdpi.com/2073-4409/9/4/916/s1, Figure S1: The relative expression levels of genes investigated in this study, Figure S2: Effects of long-term treatment with N deficiency on rice seminal root growth, Table S1: List of primers used in this research.

## Abbreviations

CK	Cytokinin
CKX	Cytokinin oxidase dehydrogenase
DZ	dihydrozeatin
EXP	Expansin
FW	Fresh weight
iP	Isopentenyladenine
N	Nitrogen
NS	Not significant
PLT	PLETHORA
XTH	Xyloglucan endotransglucosylase/hydrolase
Z	Zeatin
