# Supplementary material for: Nitrogen Deficiency-Induced Decrease in Cytokinins Content Promotes Rice Seminal Root Growth by Promoting Root Meristem Cell Proliferation and Cell Elongation
Source: Cells. 2020 Apr 9;9(4):916. doi: 10.3390/cells9040916 (PMC7226747; doi:10.3390/cells9040916)
Supplement: Supplementary file 1 [file cells-09-00916-s001.pdf]

Supplementary Materials

Supplementary Figure S1

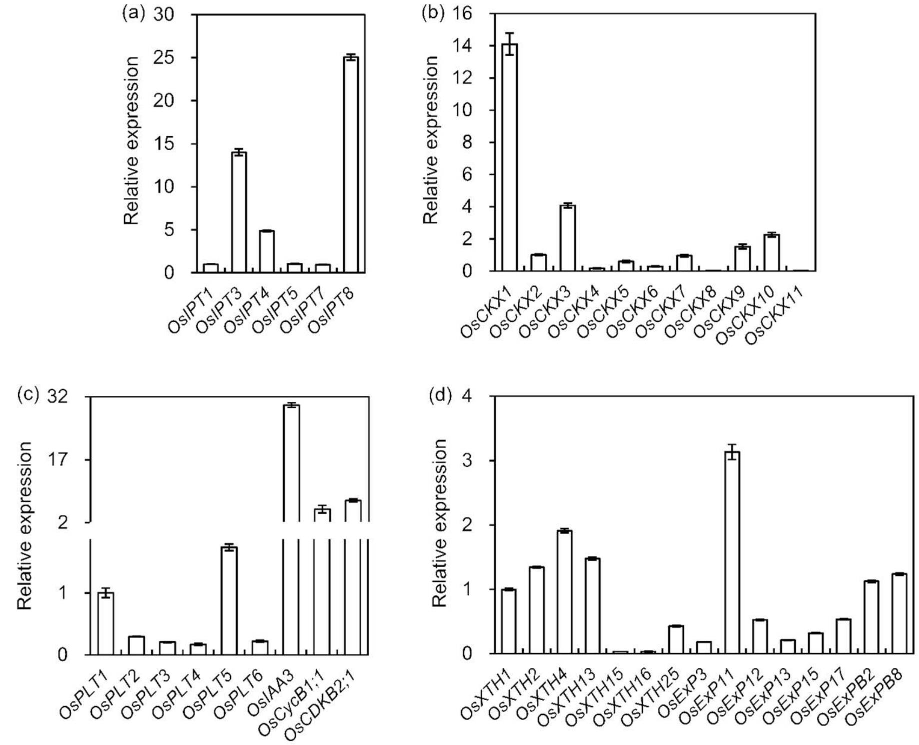

Figure 1. The relative expression levels of CK metabolism genes, root meristem size-related genes, and root cell elongation-related genes in rice seminal root. (a) Relative expression levels of CK biosynthesis genes. The relative expression level of *OsIPT1* was set as 1. (b) Relative expression levels of CK degradation genes. The relative expression level of *OsCKX2* was set as 1. (c) Relative expression levels of root meristem size-related genes. The relative expression level of *OsPLT1* was set as 1. (d) Relative expression levels of cell elongation-related genes. The relative expression level of *OsXTH1* was set as 1. Germinated rice seeds were incubated with a solution containing 1 mM N. After one day of treatment, the seminal roots of rice seedlings were sampled for qRT-PCR analyses. N, nitrogen.

Supplementary Figure S2

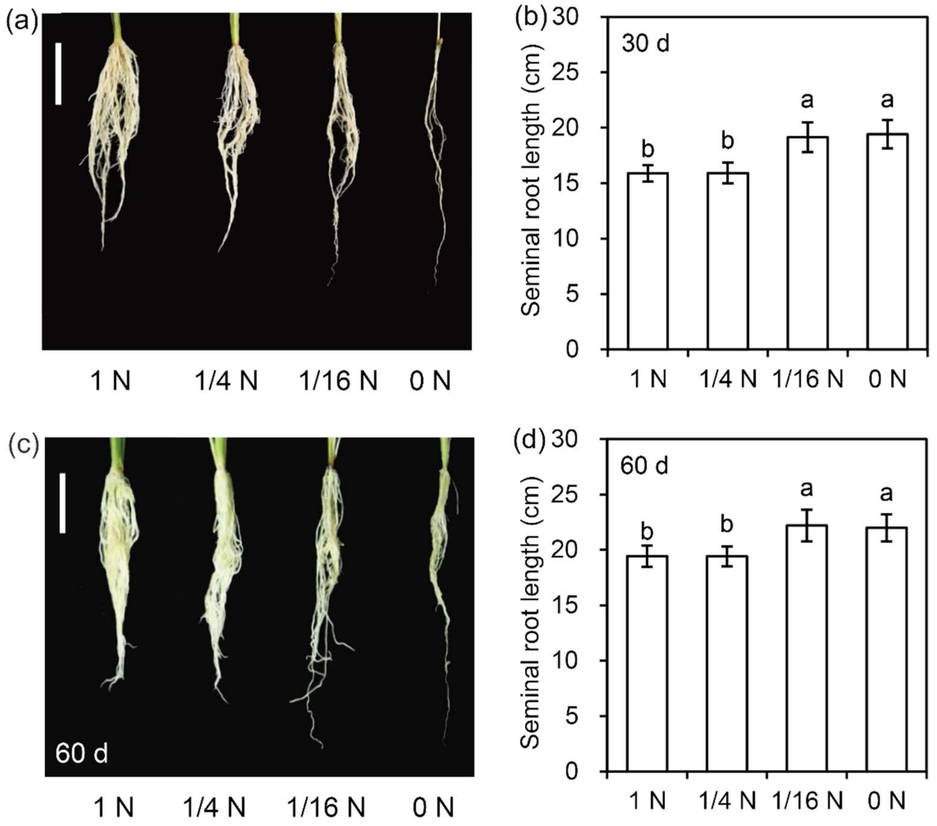

Figure 2. Effects of long-term treatment with N deficiency on rice seminal root growth. (a, b) Comparison of root phenotype and seminal root length after 30 days of different treatments. (c, d) Comparison of root phenotype and seminal root length after 60 days of different treatments. Scale bar is 5 cm for (a) and (c). In this experiment, germinated rice seeds were incubated in different solutions for 30 days or 60 days, then photographs were taken, and the lengths of the seminal roots were measured. The data are the means ± SD calculated from ten biological replicates. Significant differences ($P < 0.05$) are indicated by different letters. N, nitrogen; 1 N, 1 mM N; 1/4 N, 1/4 mM N; 1/16 N, 1/16 mM N; 0 N, 0 mM N.

Supplementary Table S1

Table 1. List of primers used in this research.

Gene name	Forward primer 5' → 3'	Reverse primer 5' → 3'
OsIPT1	AAGTCCAAGCTCGCCATCC	TCCTCGTCGGTGACCTTGTT
OsIPT3	GACAAGGGCAAGGTAGTGGT	GTCGTGCACCTGGATCTTGT
OsIPT4	CGGCGTCAAGCACAAGG	TCGTCACCACATCCAACCC
OsIPT5	CATCCCGAGGCTTGTTCC	TGCTCCTGCTGACGCTGA
OsIPT7	AGCGTTGCCACCAGGATTG	GCTGAATCTTGTCGGCGTTG
OsIPT8	CGGGTCCAACTCGCTCATC	AGGAGGCAGCACGGGAAC
OsCKX1	CAAATCCAAGTGGGATGCGG	CGACACCACGTAGAACACCTC
OsCKX2	GAACCGCAACAAGTGGGACA	TGTAGAACACCTCGTCACCG
OsCKX3	TTGGCACCAAGAACAACCCT	TGTGGCTCATCTGCCTTGAG
OsCKX4	CAAGCCAGTTCCAGTCGGAT	TGATGTCAGCCTCATCGTGG
OsCKX5	CGTCACTCGCGAGCAAATAC	CACCATGAACACCAAGCACC
OsCKX6	CAAGGAGCCGGACTTGTTCT	GTCACCACATCCGAGTAGGC
OsCKX7	ATGAGGACATCGGGTGCAAG	TGTGTGGGTCATACTTCGCC
OsCKX8	CGACAGCTTCGAGACCTTCA	TGAAGTTGAGCTGAGCAGGG
OsCKX9	CCTGGTGGCATTCCTATCCTC	GACTGCCACTCCTGTTCTGT
OsCKX10	TGTGGTGACAGGAATGGGTG	CAAACTGACCCAACCCACCT

OsCKX11	TGAGCGGGCAATCCTTC	GTGATGACGCCGAACTGG
OsPLT1	AACATTCGGCACTGAGGAGG	CTCCCGATTGGAAGGTTGCT
OsPLT2	CCGCTTGACTTCCCGTACAT	ATCAGATCCTCGCATGCCTT
OsPLT3	TGTTGCAACTCGTTTGGTCG	CCTGCCATTACCCAGCTTCC
OsPLT4	AAGAAGATAAGGCGGCTCGG	TGCAATGTACTCCTGCCTGG
OsPLT5	AGCAGTGGTTTCTCCAGAGG	GCTGAAGGTGCCCAAGTAGA
OsPLT6	TCCAGACGTCACAGGTTTCG	CGGGAGACCATGAAAGCCAT
OsCycB1;1	CAGGAACGCAAGGGAGGTAAG	GTATCGCAGCAAGAAAACCCC
OsCDKB2;1	CGCTCGTTCACTGTCCCTCT	CCACAGACCAGATGTCAACCG
OsIAA3	GCCATGTTCCTCTGCTTCTCC	CGCCGTCCTTGTCCTCGTAG
OsXTH1	ACCGCCTACTACATGTGCTC	ATGATGTAGGGCTCACCCGT
OsXTH2	TGATCGCGTTCTTCGTGGAC	TCCACAGGCTGGAGTAGAGC
OsXTH4	TCTGGAATGCCGATGACTGG	GCAAGCGATAGCCCTGTAGT
OsXTH13	GGTGTTCCGCAACTACCAGA	CCCATATGCTCGAGTAGGCG
OsXTH15	CAGTGTGACATCACGTGGGA	TTGGTCCGGAGCATACAACC
OsXTH16	TGATATGGGGCGAAGACCAC	ACTGGTCCTTGGACTGGAAC
OsXTH25	CGAGTCGGAGCAAGACAACT	CCTGAACCAGTGGCGTATGT
OsEXP3	ACATCGCCGTCTATCAAGCC	GTAGTCGTGACCGCTGATCG
OsEXP11	ACCATCACCGGCCACTCCT	CGACCCCTTCACCGACACC
OsEXP12	CCTCAACAACCAGGCCATCT	GGTGAATGTCTGGCCGAACT
OsEXP13	ACCAAAGGGTTCCTTGCATGA	ACTTGGAGCCCTTCACGTCC
OsEXP15	GGCTCCAGATCGGCATCTAC	GATCAGCACCAGCTCGAAGT
OsEXP17	TACAAGACATCGGACTGGCAC	GTCTTCGTCGAGTAGAGCGAC
OsEXPB2	TCGTCTACACCAACGACTGG	CGGGTACTGGTTGGTGTTCT
OsEXPB8	TACCCGTTCATGGGGATGAC	GGTTTCGATTGCACCTGACG
OsACTIN	CTGACGGAGCGTGGTTACTCAT	TCATAGTCCAGGGCGATGTAGG
